# Self-Assembled Nanoparticles of Silicon (IV)–NO Donor Phthalocyanine Conjugate for Tumor Photodynamic Therapy in Red Light

**DOI:** 10.3390/pharmaceutics16091166

**Published:** 2024-09-04

**Authors:** Kadireya Aikelamu, Jingya Bai, Qian Zhang, Jiamin Huang, Mei Wang, Chunhong Zhong

**Affiliations:** Xinjiang Key Laboratory of Natural Medicines Active Components and Drug Release Technology, Ministry of Education, Engineering Research Center of Xinjiang and Central Asian Medicine Resources, College of Pharmacy, Xinjiang Medical University, Urumqi 830017, China; 18699977617@163.com (K.A.);

**Keywords:** nitric oxide, photosensitizer, self-assembled nanoparticles

## Abstract

The combination of photodynamic therapy (PDT) and pneumatotherapy is emerging as one of the most effective strategies for increasing cancer treatment efficacy while minimizing side effects. Photodynamic forces affect nitric oxide (NO) levels as activated photosensitizers produce NO, and NO levels in the tumor and microenvironment directly impact tumor cell responsiveness to PDT. In this paper, 3-benzenesulfonyl-4-(1-hydroxy ether)-1,2,5-oxadiazole-2-oxide NO donor–silicon phthalocyanine coupling (SiPc–NO) was designed and prepared into self-assembled nanoparticles (SiPc–NO@NPs) by precipitation method. By further introducing arginyl-glycyl-aspartic acid (RGD) on the surface of nanoparticles, NO-photosensitizer delivery systems (SiPc–NO@RGD NPs) with photo-responsive and tumor-targeting properties were finally prepared and preliminarily evaluated in terms of their formulation properties, NO release, and photosensitizing effects. Furthermore, high reactive oxygen species (ROS) generation efficiency and high PDT efficiency in two breast cancer cell lines (human MCF-7 and mouse 4T1) under irradiation were also demonstrated. The novel SiPc–NO@RGD NPs show great potential for application in NO delivery and two-photon bioimaging-guided photodynamic tumor therapy.

## 1. Introduction

Cancer is a continuing public health challenge faced by the world. As the second most common cause of death in the world, cancer is expected to become the most significant barrier to increasing life expectancy in the 21st century [1,2]. According to the latest assessment by the International Agency for Research on Cancer (IARC), there will be 20 million new cancer cases globally in 2022. Among women, the most common cancer and cause of cancer death is usually breast cancer [3,4]. At the present stage, clinical treatments for breast cancer include chemotherapy, hormone therapy, targeted biotherapy, and radiation therapy [5]. However, the side effects and drug resistance caused by chemotherapeutic drugs also reduce the effectiveness of traditional chemotherapeutic drugs in treating cancer during clinical use. Finding novel, efficient targeted therapeutics based on modifications in tumor cell biology is therefore critically needed [6]. As a new treatment method, photodynamic therapy (PDT) shows potential advantages.

Photodynamic therapy (PDT) utilizes photosensitizers (PS) and specific wavelengths of light to interact with oxygen to generate cytotoxic reactive oxygen species (ROS), which trigger apoptosis and/or necrosis in the target tissue [7]. PDT has been used clinically to treat cancer for more than 40 years [8]. In 1978, Dougherty et al. found that intravenous injection of a photosensitizer called hematoporphyrin derivative (HPD) enabled a range of skin and subcutaneous tumors to be effectively treated by performing red light irradiation [9]. A photosensitizer is a substance that can absorb light energy and transfer it to another molecule, causing a photochemical reaction [10]. With the participation of oxygen, photoactivated photosensitizers produce ROS, and ROS are highly cytotoxic to tumor cells. The main ROS produced in PDT is singlet oxygen (^1^O_2_), which is formed by energy transfer in the triplet state (type II) of photosensitizers, and free radical species, such as superoxide ions (O_2_^•–^) and hydroxyl radicals (OH^•^), formed by electron transfer reactions (type I). These ROS have a very short lifespan and a small range of action. The antitumor effect of PDT is produced by the combination of three main effects: (a) direct cytotoxic effects on cancer cells, (b) destruction of tumor blood vessels, and (c) stimulation of antitumor immunity [11]. Photosensitizers, as one of the key components of photodynamic therapy, have been increasingly emphasized and researched, and the existing photosensitizers have been categorized into three generations based on their properties and the direction of their development. HPD was the first photosensitizer to be used in photodynamic therapy, and it is grouped with porphyrin derivatives such as dihematoporphyrin ether and Photofrin. Second-generation photosensitizers, including methylene blue [12] and porphyrins [13,14], have better selectivity and are able to aggregate more accurately in tumor tissues, but their disadvantages of unstable therapeutic efficacy, inhomogeneous biodistribution, toxicity, and side effects require further research to enhance their therapeutic efficacy and reduce adverse effects. As a research hotspot in recent years, most of the third-generation photosensitizers are based on the introduction of various functional groups on the basis of known photosensitizers, such as targeting groups, chemotherapeutic drug groups, biocompatibility groups, and stability-enhancing groups, which, compared with earlier photosensitizers, have the advantages of high specificity [15], high photosensitivity stability [16], high photoconductivity [17], good tissue penetration [18], and the ability to be combined with nanotechnology [19].

Most PDT agents absorb light in the visible range (wavelengths of 400–700 nm) [20] or near-infrared (NIR) range (700–1350 nm) [21]. Longer wavelengths of near-infrared (NIR) light minimize the extent of tissue scattering and penetrate deeper than visible light, resulting in a wider range of applications. Recently, in addition to NIR light, X-ray radiation has emerged as a promising source of energy that is capable of more effective PDT for deep tumors [22]. In addition to external excitation sources, PDT reagents can be designed to be excited by enzyme-mediated bioluminescence methods, thus overcoming optical depth limitations [23].

Nitric oxide (NO) as a gaseous signaling molecule has multiple biological functions. Low concentrations of NO promote a variety of signaling pathways, thereby promoting tumor development and invasion. However, high NO concentrations promote DNA damage, protein dysfunction, gene mutation, and cell death [24]. Therefore, to facilitate the precise delivery and controlled release of NO, a variety of low molecular NO donors have been developed, such as diazeniumdiolates (NONOates) [25], S-nitrosothiols (RSNO) [26], and oxidized furans (furoxans) [27]. In addition to acting directly on cells or tissues, NO can also have a sensitizing effect on other therapeutic modalities. Despite possessing considerable significant advantages, NO-based cancer therapy is still limited by short half-life and off-target effects [28].

Recently, researchers have turned to self-assembled nanoparticles (SANs) to design stimuli-responsive and targeted self-assembled nanoplatforms by taking advantage of their small particle size, high drug loading capacity, and the ability to achieve controlled release of drugs, thereby increasing efficacy and reducing toxic side effects [29]. Therefore, it is highly desirable to utilize the above-mentioned factors to prepare biocompatible self-assembled nanoparticles that produce both NO and ROS for more effective photodynamic therapy.

**Figure 1:**
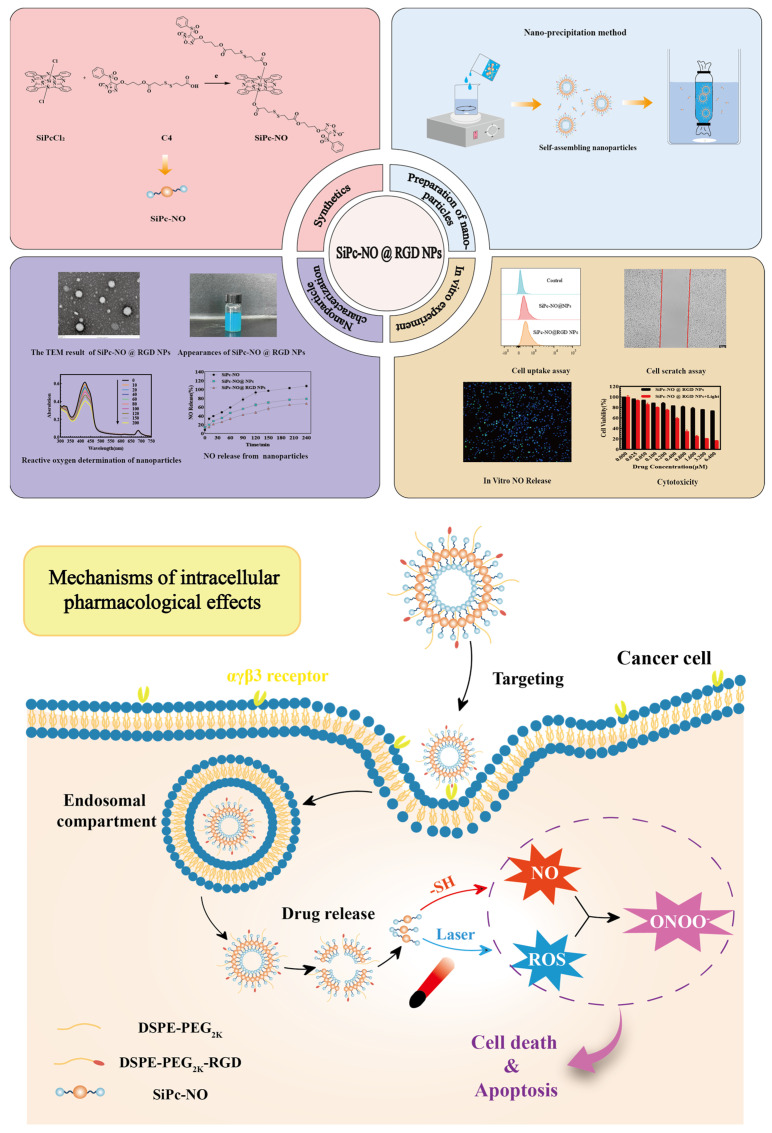
Schematic of the preparation, characterization and anticancer effect of SiPc-NO@RGD NPs.

Therefore, in this study, the drug–drug conjugate was prepared to be constructed by connecting NO donor (3-benzenesulfonyl-4-(1-hydroxy ether)-1,2,5-oxadiazole-2-oxide) and silicon phthalocyanine photosensitizer, and self-assembled into nanoparticles (SiPc–NO@NPs) in water under the presence of 1,2-distearoyl-sn-glycero-3-phosphoethanolamine-N-[methoxy (polyethylene glycol)-2000] (DSPE-PEG_2K_). The arginine-glycine-aspartate (RGD) peptide was further introduced on the surface of the nanoparticles, which could specifically bind to 11 integrins and improve their targeting, and finally a photosensitive and tumor-targeted drug delivery system was prepared, as shown in detail in Figure 1. In vitro PDT studies demonstrated that SiPc–NO@NPs and SiPc–NO@RGD NPs performed well in imaging-guided photodynamic tumor therapy. This study addresses the shortcomings of gas therapy and photodynamic therapy alone, improves the water solubility of SiPc photosensitizer, and achieves effective delivery of NO, laying a theoretical foundation for synergistic treatment with gas therapy and photodynamic therapy.

## 2. Materials and Methods

### 2.1. Materials and Equipment

(Phenylthio)acetic acid and 3,3′-dithiodipropionic acid were purchased from Aladdin Biochemical Technology Company (Shanghai, China). 3-chloroperoxybenzoic acid, 1,3-propanediol, and 1-(3-dimethylaminopropyl)-3-ethylcarbodiimide hydrochloride (EDC) were obtained from Macklin Chemical Reagent Company (Shanghai, China). Silicon phthalocyanine dichloride (SiPcCl_2_) was purchased from Bide Pharmaceutical Technology Company (Shanghai, China). DSPE-mPEG_2K_-RGD was obtained from Xi’an Ruixi Biotechnology Company (Xi’an, China). DSPE-PEG_2K_ was purchased from AVT Pharmaceutical Technology Company (Shanghai, China). 1,3-diphenylisobenzofuran (DPBF) was purchased from Maokang Biotechnology Company (Shanghai, China). Griess reagent was purchased from Beyotime Biotechnology (Shanghai, China). Cell Counting Kit-8 (CCK-8) was obtained from AbMole Bioscience Company (Houston, TX, USA). 2,7-dichlorodihydrofluorescein diacetate (DCFH-DA) was purchased from Solarbio Science and Technology Company (Beijing, China). 4-amino-5-(N-methylamin)-3′,6′-bis(acetyloxy) (DAF-FM-DA) was obtained from KeyGEN BioTECH Company (Nanjing, Jiangsu, China). Annexin V-FITC/PI Apoptosis Detection Kit was purchased from 4A Biotechnology Company (Beijing, China). The 4T1 breast cancer cells were supplied by iCell Bioscience Inc. (Shanghai, China).

The obtained mesoporous materials were structurally characterized by Fourier-transform infrared spectroscopy (FTIR). ^1^H-NMR and ^13^C-NMR spectra were recorded on a Unity-Inova600 NMR spectrometer. High-resolution mass spectra were recorded on a Thermo Scientific QE plus a high-resolution mass spectrometer. Electronic absorption spectra were measured on a UV-2700 spectrophotometer (Shimadzu, Co., Ltd., Kyoto, Japan), and fluorescence spectra were taken on an RF-5301PC fluorescence spectrometer (Shimadzu, Co., Ltd., Kyoto, Japan).

### 2.2. Synthesis of 4-(3-Hydroxypropoxy)-3-(phenylsulfonyl)-1,2,5-oxadiazole 2-Oxide (NO Donor)

The NO donor (**C3**) was synthesized in three steps [30]. To a stirred solution of phenylthioacetic acid (5.98 mmol, 1 g) in CH_2_Cl_2_ (5 mL), 3-chloroperoxybenzoic acid (14.95 mmol, 2.58 g) was added under a nitrogen atmosphere while maintaining an ice bath. After stirring the mixture at room temperature for approximately 1 h, the reaction was quenched with a Na_2_SO_3_ solution. The aqueous layer was extracted with CH_2_Cl_2_ (20 mL × 3). The combined extracts were washed with brine (30 mL), dried over anhydrous Na_2_SO_4_, filtered, and concentrated in vacuo. The phenylsulfonylacetic acid (**C1**) obtained was used for the next step without further purification. Yield: 1.149 g, 96%. The obtained **C1** (12.5 mmol, 2.5 g) was dissolved in acetic acid (7.5 mL), followed by the addition of 65–68% dilute nitric acid (187.5 mmol, 11.8 g). The reaction mixture was stirred at 90 °C for about 1.5 h. The mixture was cooled to room temperature and a white precipitate was precipitated by the addition of water. The precipitate was filtered and dried to obtain 3,4-diphenylsulfonyl-1,2,5-oxadiazole-2-oxide (**C2**). Yield: 1.838 g, 80.4%. To a solution of **C2** (2.73 mmol, 1 g) and 1,3-propanediol (16.38 mmol, 1.245 g) in THF (5 mL) was added 25% solution of NaOH 0.48 mL at room temperature. The reaction proceeded under a nitrogen atmosphere for 15 min until TLC indicated the complete consumption of the starting material. The mixture was diluted with water 20 mL, and extracted with EtOAc. The combined organic layers were washed with brine (20 mL), dried over Na_2_SO_4_, and evaporated under reduced pressure. The residue was purified by column chromatography on silica gel using CH_2_Cl_2_–EtOAc as the eluent to give the desired product **C3**. Yield: 0.471 g, 57.5%. IR/cm^−1^: 620, 994, 1040, 1153, 1442, 1550, 1616, 3097, 3365. ^1^H-NMR (600 MHz, CDCl_3_) δ 8.08–8.03 (m, 2H), 7.76 (td, *J* = 7.5, 1.3 Hz, 1H), 7.67–7.60 (m, 2H), 4.60 (t, *J* = 6.0 Hz, 2H), 3.89 (t, *J* = 5.8 Hz, 2H), 4.60 (t, *J* = 5.0 Hz, 2H) 7.67–7.60 (m, 2H), 4.60 (t, *J* = 6.0 Hz, 2H), 3.89 (t, *J* = 5.8 Hz, 2H), 2.13 (q, *J* = 5.9 Hz, 2H). ^13^C-NMR (151 MHz, DMSO-d6) δ 163.50, 138.75, 133.47, 138.68, 127.48, 128.68, 127.48, 128.68, 127.48, 128.68, 127.48, 127.48, 127.48, 127.48, 127.48 128.68, 127.48, 59.43.

### 2.3. Synthesis of SiPc–NO (C5)

A solution of **C3** (1 g, 3.333 mmol)**,** DTDP (2.1 g, 9.99 mmol), EDC (0.884 mL, 4.99 mmol), and DMAP (66 mg, 0. 533 mmol) in CH_2_Cl_2_ (20 mL) was stirred at room temperature under nitrogen atmosphere for 48 h [30]. The mixture was diluted with CH_2_Cl_2_ (20 mL), washed with brine (20 mL), dried over anhydrous Na_2_SO_4_, and filtered and concentrated in vacuo. The residue was purified by column chromatography on silica gel using CH_2_Cl_2_–CH_3_OH as the eluent to give the desired product **C4**. Yield: 1.044 g, 63.7%.

To a solution of **C4** (0.482 g, 0.981 mmol) in toluene (10 mL) was added SiPcCl_2_ (0.200 g, 0.327 mmol) at room temperature. The reaction proceeded under a nitrogen atmosphere at 120 °C for 36 h until TLC indicated the complete consumption of the starting material [31]. After the reaction was completed, the reaction system was filtered, and the filtrate was washed with brine (5 mL × 2) and dried over Na_2_SO_4_. The mixture was removed under reduced pressure, and the residue was purified by column chromatography on silica gel using PE:EA:CH_3_OH as the eluent to give the desired product SiPc–NO (**C5**). Yield: 0.199 g, 40%, with the following characteristics: IR/cm^−1^: 1733, 1550, 1334, 1079, 685. ^1^H-NMR (600 MHz, DMSO-d6) δ 9.70 (dp, *J* = 6.6, 3.7, 3.2 Hz, 8H), 8.54 (dt, *J* = 5.8, 3.3 Hz, 8H), 7.99–7.95 (m, 4H), 7.82 (tt, *J* = 7.2, 1.3 Hz, 2H), 7.67 (t, *J* = 8.0 Hz, 4H), 4.29 (t, *J* = 6.2 Hz, 4H), 3.97 (t, *J* = 6.3 Hz, 4H), 1.97 (dt, *J* = 20.2, 6.5 Hz, 4H), 1.89 (t, *J* = 20.2, 6.5 Hz, 4H), 1.17 (t, *J* = 7.3 Hz, 4H), 0.42 (t, *J* = 6.4 Hz, 4H), −0.27 (t, *J* = 6.0 Hz, 4H). ^13^C-NMR (151 MHz, DMSO-d6) δ 168.51, 168.14, 161.72, 155.82, 155.69, 147.08, 134.88, 132.65, 132.50, 131.39, 128.44, 126.69, 126.71, 125.53, 121.00, 120.50, 107.36, 64.93, 64.71, 57.64, 57.19, 30.92, 30.59, 30.22, 29.96, 29.45, 28.72, 26.57, 24.71, 24.83, −2.00, −2.99. HRMS (APCI) for C_66_H_55_N_12_O_18_S_6_Si (*m*/*z*): 1523.18. Found 1523.18 [M + H] ^+^.

### 2.4. Preparation and Characterization of Self-Assembled NPs

#### 2.4.1. Preparation of Self-Assembled NPs

All nanoparticles were prepared using the nanoprecipitation method [32]. At room temperature, 1 mL of tetrahydrofuran (THF) solution of SiPc–NO (2.5 mg/mL) and DSPE-PEG_2K_ (1 mg/mL) was injected into 3 mL of purified water with vigorous stirring. The aqueous solution turned light blue while self-assembling into nanoparticles. Unself-assembled SiPc–NO and THF were removed by dialysis. The concentration of SiPc–NO in NP solution was determined by high-performance liquid chromatography (HPLC, Shimadzu, LC-20A) with a UV–Vis detector. For RGD-modified nanoparticles, DSPE-PEG_2K_ (0.5 mg) and DSPE-PEG_2K_-RGD (0.5 mg) were dissolved in 1 mL of SiPc–NO (2.5 mg/mL) THF solution. The mass ratio of SiPc–NO to DSPE-PEG_2K_ (RGD) was controlled at 5:2.

#### 2.4.2. Characterization of Self-Assembled NPs

Particle size, PDI, and zeta potential of SiPc–NO@NPs and SiPc–NO@RGD NPs were determined using a Zetasizer particle sizer (Zetasizer Nano-ZS, Malvern, UK).

Nanoparticle morphology was observed using a transmission electron microscope (JEM-1400 plus, JEOL, Tokyo, Japan).

The encapsulation efficiency (EE) and drug loading (DL) of SiPc–NO in nanoparticles were determined by HPLC. EE and DL were calculated using the following formulas:

$$\mathrm{DL}\left(\mathrm{wt}\%\right)=\frac{\mathrm{the}\;\mathrm{weight}\;\mathrm{of}\;\mathrm{SiPc}-\mathrm{NO}\;\mathrm{in}\;\mathrm{NPs}}{\mathrm{the}\;\mathrm{weight}\;\mathrm{of}\;\mathrm{total}\;\mathrm{NPs}}\times 100\%$$

$$\mathrm{EE}\left(\mathrm{wt}\%\right)=\frac{\mathrm{the}\;\mathrm{weight}\;\mathrm{of}\;\mathrm{SiPc}-\mathrm{NO}\;\mathrm{in}\;\mathrm{NPs}}{\mathrm{the}\;\mathrm{weight}\;\mathrm{of}\;\mathrm{feeding}\;\mathrm{SiPc}\;-\mathrm{NO}}\times 100\%$$

### 2.5. Stability of Self-Assembled NPs

To assess the stability of NPs, SiPc–NO@NPs and SiPc–NO@RGD NPs were stored at room temperature and 4 °C for 30 days. Particle sizes, PDI, and zeta potential were measured at different times (1, 3, 5, 8, 14, 22, and 30 d) to examine the storage stability of the nanoparticles [33].

### 2.6. In Vitro NO Release

In vitro NO release studies were performed using the Griess method [34]. The concentrations of SiPc–NO, SiPc–NO@NPs, and SiPc–NO@RGD NP solutions were adjusted to 40 μM, and 50 μL was taken at different time points (0, 20, 40, 60, 80, 100, 120, 150, 180, 210, and 240 min) and mixed with 50 μL of the Griess I and II reagents. The solutions were kept at room temperature for 10 min and then measured at 540 nm using a microplate reader. The NO concentration was calculated using the regression equation of the NaNO_2_ standard solution.

Intracellular detection of NO release from 4T1 and MCF-7 cells was performed with the DAF-FM-DA fluorescent probe [35]. The 4T1 and MCF-7 cells were inoculated in 24-well plates (7 × 10^4^/well). After 24 h of incubation, the cells were incubated with NPs for 5 h, and then 5 μM DAF-FM-DA was added and incubated for 1 h. After DAPI staining and 4% PFA fixation, fluorescence images of NO release from 4T1 cells were observed by fluorescence inverted microscope at excitation and emission wavelengths of 495 nm and 515 nm, respectively.

### 2.7. Cell Counting Kit-8 Assay

The photodynamic therapy efficacy of SiPc–NO self-assembled nanoparticles against 4T1 and MCF-7 cells was determined by CCK-8 assay [36,37]. The 4T1 and MCF-7 cells were inoculated into 96-well plates at densities of 10^4^ and 1.3 × 10^4^ per well, respectively, and cultured for 24 h. Different concentrations (0, 0.025, 0.05, 0.1, 0.2, 0.4, 0.8, 1.6, 3.2, 6.4 μM) of SiPc–N@NPs and SiPc–NO@RGD NPs in serum-free medium solutions were added and a blank control group was established. Subsequently, after 48 h of incubation under light-free conditions, the well plates were processed according to the CCK-8 method.

In the light group, cells were irradiated with 680 nm laser (95.5 mW/cm^2^) for 1.5 min/well after 3 h of drug administration, and then the OD value at 450 nm was measured. Cell survival was calculated by the following formula:

$$\mathrm{Cell}\;\mathrm{viability}\left(\%\right)=\frac{{\mathrm{OD}}_{\mathrm{sample}}-{\mathrm{OD}}_{\mathrm{blank}}}{{\mathrm{OD}}_{\mathrm{control}}-{\mathrm{OD}}_{\mathrm{blank}}}\;\times \;100\%$$

### 2.8. Intracellular Uptake

#### 2.8.1. Laser Confocal Scanning Microscope Method

The 4T1 and MCF-7 cells were inoculated at a density of 5 × 10^4^ cells per cell in a 35 cm^2^ four-cell confocal Petri dish, and incubated for 24 h at 37 °C in 5% CO_2_. After discarding the medium, serum-free mediums containing SiPc–NO@NPs and SiPc–NO@RGD NPs were added and co-cultured with cells for 3 h, respectively, and then washed three times with 1 mL PBS. They were stained with DAPI for 10 min and washed three times with 1 mL PBS, and their uptake was observed using a laser confocal microscope [38,39].

#### 2.8.2. Flow Cytometry Method

When the cells were in the logarithmic growth phase, 5 × 10^5^ cells/well were inoculated into 6-well plates and incubated in an incubator with 5% CO_2_ and saturated humidity at 37 °C for 24 h. After the cells adhered to the wall, the culture medium was discarded. SiPc–NO@NPs and SiPc–NO@RGD NPs were added to each group of cells (a blank control group was set up) and co-cultured with cells for 3 h. At the end of incubation, the cells were washed with PBS three times, digested with trypsin, and collected, and the fluorescence intensity was measured within 1 h.

### 2.9. Reactive Oxygen Species Generation Ability

The 4T1 and MCF-7 cells were inoculated at a density of 3 × 10^5^ cells per dish in 35 mm^2^ confocal culture dishes for 24 h. The cells were co-incubated with SiPc–NO@NPs and SiPc–NO@RGD NPs for 5 h. After that, the light group was irradiated with a 680 nm laser (95.5 mW/cm^2^) for 10 min, after which the culture medium was discarded and washed three times with 0.5 mL of PBS, and 10 μM of DCFH-DA solution was added, incubated for 30 min, and washed three times with 0.5 mL of PBS. The production of reactive oxygen species in the cells was then observed under fluorescent inverted microscope for SiPc–NO@NPs and SiPc–NO@RGD NPs, respectively [40].

### 2.10. Scratch Assay

The effect of SiPc–NO self-assembled nanoparticles on the migration of breast cancer cells was investigated by cell scratch assay. The cells were inoculated into 12-well plates at 1.5 × 105 cells/well and incubated in an incubator overnight. After the cell density reached over 90%, a line was drawn in the center of the plate wells by sliding the 200 μL tip vertically, and then the cells suspended on the scratch were washed three times with PBS. Then, serum-free medium with SiPc–NO @NPs and SiPc–NO@RGD NP was added for the experimental group, while l mL of serum-free medium was added for the blank control group, respectively. The treated cells were placed in an incubator for further incubation, and three randomly selected points within the scratch area were photographed at 0 h and 24 h of scratching, and the healing rate of the scratches was calculated using ImageJ software (version v1.53t.) [41].

$$\mathrm{Migration}\;\mathrm{area}\left(\%\right)=\frac{\mathrm{Initial}\;\mathrm{Scratch}\;\mathrm{Area}-\mathrm{Scratch}\;\mathrm{Area}\;\mathrm{after}\;24\;h}{\mathrm{Initial}\;\mathrm{Scratch}\;\mathrm{Area}}\times 100\%$$

## 3. Results

### 3.1. Synthesis and Characterization

**Figure 2:**
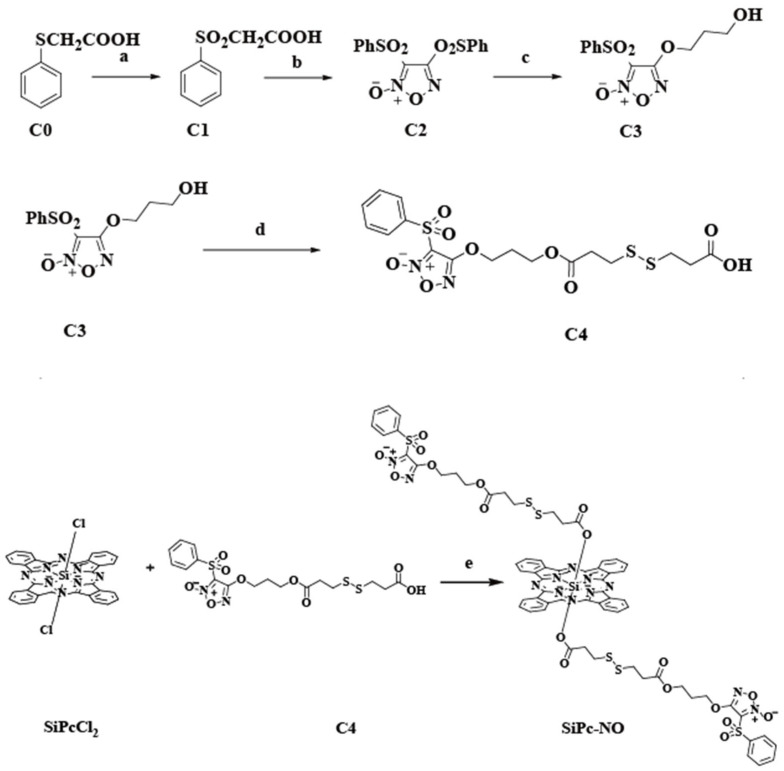
Synthesis routes of the SiPc–NO. (a) m-CPBA, DCM, r.t., 1 h; (b) CH_3_COOH, nitric acid, reflux, 1.5 h; (c) 1,3-propanediol, NaOH, THF, r.t., 15 min; (d) DTDP, EDC, DMAP, DCM, r.t., 48 h; (e) toluene, reflux, 36 h.

The synthetic scheme of SiPc–NO is shown in Figure 2. The analytical methods such as UV, IR, NMR (^1^H-NMR, ^13^C-NMR), and HRMS were used to characterize SiPc–NO, and the feasibility of the synthetic pathway was further confirmed by comparing the changes in the characteristic functional groups of the raw materials and products. SiPc–NO is soluble in several commonly used solvents such as dichloromethane (CH_2_Cl_2_), dimethyl sulfoxide (DMSO), N,N-dimethyl formamide (DMF), and tetrahydrofuran (THF). The ^1^H NMR spectrum of SiPc–NO is shown (Supplementary Figure S1). Two sets of resonances were observed at 9.7 and 8.54 ppm for the eight silicon phthalocyanine protons. The double resonances for axially substituted aromatic protons appeared at 7.99, 7.82, and 7.67 ppm. In the IR spectra, the characteristic –COOH peak of C4 disappears after the reaction in step five (Supplementary Figure S3). In addition, the HRMS spectrum showed a strong single-charged molecular ion peak for the product, which is consistent with the proposed structure (Supplementary Figure S4).

### 3.2. Preparation and Characterization of Self-Assembled NPs

**Figure 3:**
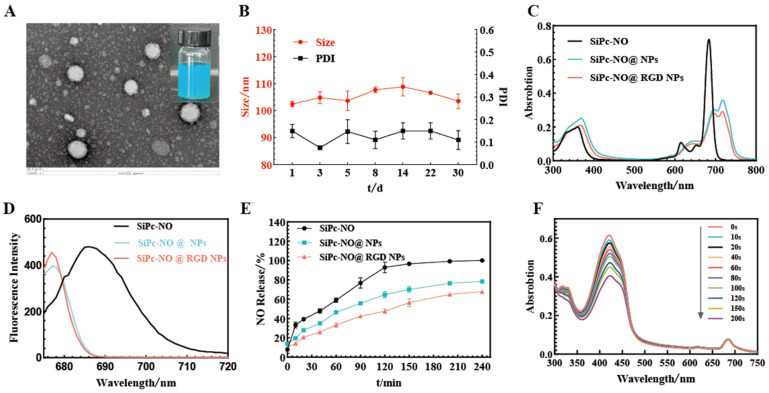
(**A**) The TEM result and appearances of SiPc–NO@RGD NPs. (**B**) Stability of SiPc–NO@RGD NPs in water stored at room temperature for 30 days. (**C**) UV spectrum of SiPc–NO self-assembled nanoparticles. (**D**) Fluorescence spectra of SiPc–NO self-assembled nanoparticles. (**E**) NO release from SiPc–NO self-assembled nanoparticles. (**F**) Reactive oxygen determination of SiPc–NO@RGD NPs.

The morphology of SiPc–NO@RGD NPs was investigated by transmission electron microscopy. SiPc–NO@RGD NPs were spherical with an average diameter of about 90 nm and hydrodynamic size of about 100 nm, and were highly dispersible in water (Figure 3A). The average size of SiPc–NO@RGD NPs observed by electron microscopy was smaller than the average size observed in water, which could be a result of the swelling of nanoparticles in water.

The size distributions of SiPc–NO@NPs and SiPc–NO@RGD NPs nanoparticles remained stable for 30 days, demonstrating their excellent stability at room temperature and 4 °C (Figure 3B). HPLC analysis showed minimal SiPc–NO precipitation after nanoparticle formation. As a result, the SiPc–NO encapsulation efficiency was 80%, with a drug loading capacity exceeding 60%, establishing a solid foundation for effective NO delivery and photodynamic therapy.

### 3.3. Photophysical and Photochemical Properties

The UV–Vis spectrum of SiPc–NO in THF is shown in Figure 3C. SiPc–NO observed a B-band around 360 nm and a typical unaggregated Q-band at 680 nm. As the concentration of SiPc–NO increased, there was no significant change in the position of the B band, and the absorbance of the Q band showed a good linear relationship with the concentration. To improve the water solubility and targeting of SiPc–NO, it was self-assembled into SiPc–NO@NPs and SiPc–NO@RGD NPs in aqueous solutions containing DSPE-PEG_2k_ and DSPE-PEG_2k_-RGD. The two self-assembled nanoparticles were well dispersed in water and the Q-band was located at 718 nm, which was 38 nm red-shifted from the Q-band of SiPc–NO in THF. The Q-band redshift was attributed to the interaction of SiPc–NO with DSPE. The reduced uptake of both self-assembled nanoparticles in water compared to SiPc–NO was attributed to the aggregation of SiPc–NO in the nanoparticles.

As depicted in Figure 3D, SiPc–NO, SiPc–NO@NPs, and SiPc–NO@RGD NPs exhibited intense fluorescence emission signals at 690 nm when excited at 675 nm, with the emission wavelengths of the different dosage groups in water being blue-shifted in comparison to SiPc–NO.

Furan-based nitric oxide donors are conventional NO donors with potential applications in antitumor therapy, efficiently releasing NO in vivo in response to thiols or enzymes [42]. The NO release from SiPc–NO, SiPc–NO@NPs, and SiPc–NO@RGD NPs was measured using the Griess method, with the results shown in Figure 3E. After 3 h, the releases of SiPc–NO, SiPc–NO@NPs, and SiPc–NO@RGD NPs were approximately 99%, 73%, and 62%, respectively, followed by a gradual release. Within 4 h, the free SiPc–NO could release 100% of NO, whereas the dosage from the two nanoparticle groups could only release about 60–80%. This difference may be attributed to the nanoparticles compacting the drug, leading to a slower release rate as they had to overcome the obstruction of the nanocarrier surface layer.

The reactive oxygen yields of SiPc–NO, SiPc–NO@NPs, and SiPc–NO@RGD NPs were evaluated by UV–Vis spectroscopic analysis using 1,3-diphenylisobenzofuran (DPBF) as scavenger (Supplementary Figure S5). The results of SiPc–NO@RGD NPs are shown in Figure 3F. When SiPc–NO@RGD NPs were introduced into the DPBF solution, the absorbance of the DPBF fluorescent probe at 415 nm notably decreased, suggesting that the SiPc–NO@RGD NPs could create reactive oxygen species efficiently when exposed to laser irradiation (680 nm, 95.5 mW/cm^2^).

### 3.4. Cytotoxicity

**Figure 4:**
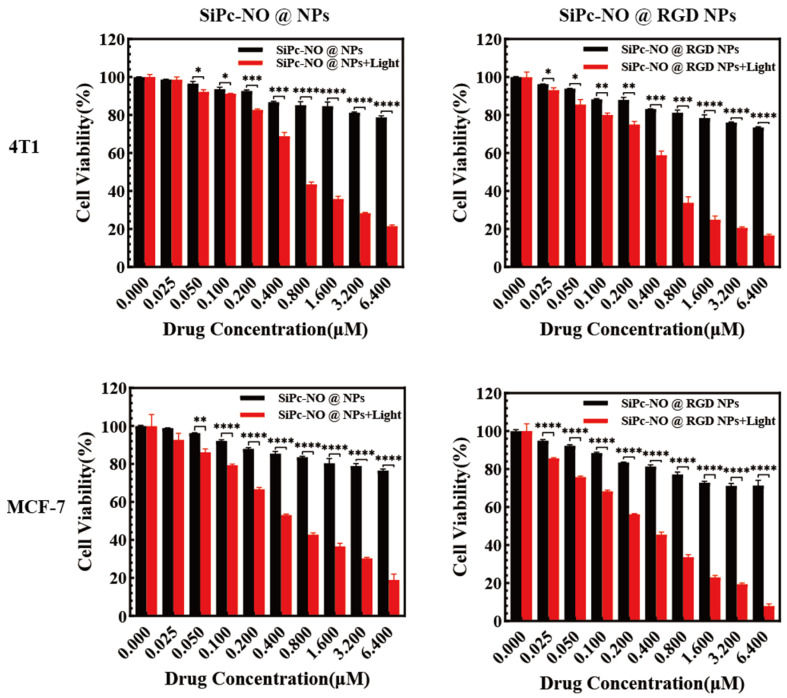
Cytotoxicity of SiPc–NO self-assembled nanoparticles. Significance identification: *, *p* < 0.05; **, *p* < 0.01; ***, *p* < 0.001, ****, *p* < 0.0001.

Data on SiPc–NO were unavailable due to significant aggregation and precipitation within a brief timeframe. Figure 4 demonstrates the antiproliferative impact of the self-assembled SiPc–NO nanoparticles on 4T1 and MCF-7 breast cancer cells. The survival rate of cancer cells under the absence of laser irradiation conditions showed a slight decrease with an increasing drug concentration of SiPc–NO@NPs and SiPc–NO@RGD NPs, but they were all above 75%. This indicated that SiPc–NO@NPs and SiPc–NO@RGD NPs have good biocompatibility in the cells.

Upon irradiation with a 680 nm laser (95.5 mW/cm^2^), the survival rate of the two types of cells decreased significantly with the increase of the concentration of SiPc–NO self-assembled nanoparticles, and the phototoxicity of SiPc–NO@RGD NPs was stronger than that of SiPc–NO@NPs on 4T1 and MCF-7 cells, which was a significant difference. The IC_50_ values of SiPc–NO@NPs and SiPc–NO@RGD NPs on 4T1 and MCF-7 cells were analyzed and the results are shown in Table 1.

### 3.5. Cell Uptake Assay

**Figure 5:**
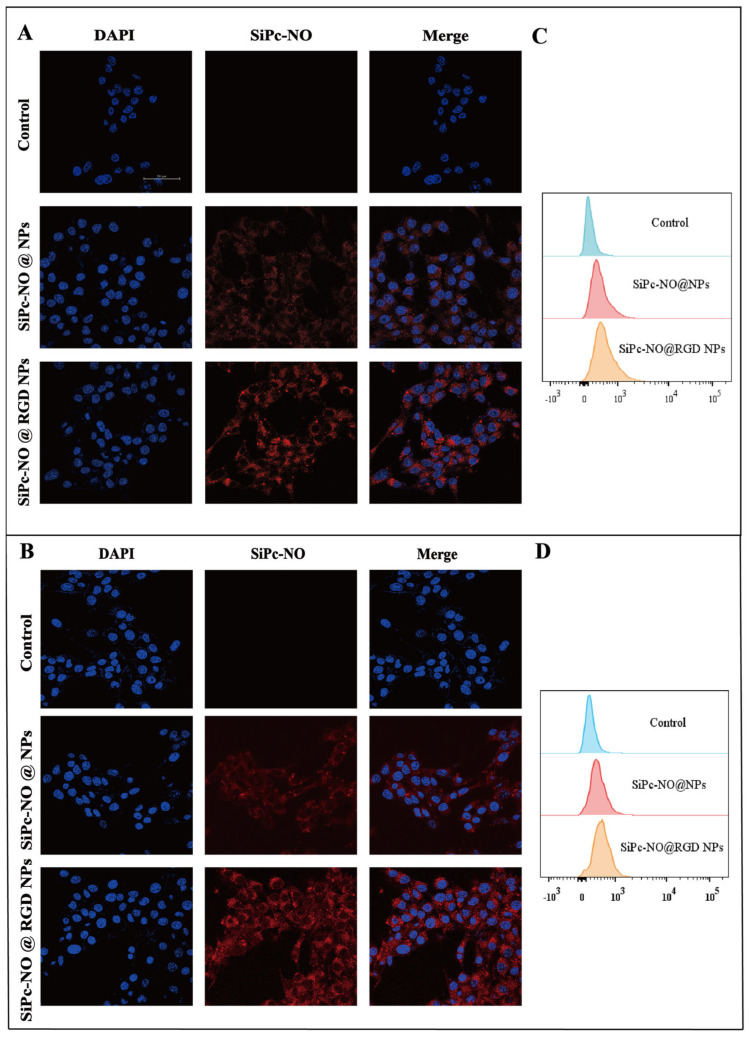
(**A**) Cellular uptake of NPs in 4T1 cells detected by CLSM. (**B**) Cellular uptake of NPs in MCF-7cells detected by CLSM. (**C**) Cellular uptake of NPs in 4T1 cells detected by flow cytometry. (**D**) Cellular uptake of NPs in MCF-7 cells detected by flow cytometry.

The uptake of SiPc–NO self-assembled nanoparticles by cells is crucial for assessing their photodynamic therapeutic potential. To evaluate this, 4T1 and MCF-7 cells were co-cultured with SiPc–NO@NPs and SiPc–NO@RGD NPs for 3 h, and the cellular uptake of the nanoparticles was observed using CLSM. The results are depicted in Figure 5A,B, with red fluorescence observed in the cells after 3 h of administration, indicating that both SiPc–NO@NPs and SiPc–NO@RGD NPs were efficiently internalized by the cells. In addition, SiPc–NO@RGD NPs were taken up more by 4T1 and MCF-7 cells, showing the most intense red fluorescence. In contrast, SiPc–NO@NPs lacking RGD modifications were less readily taken up by the cells, highlighting the targeting ability of the self-assembled nanoparticles.

The flow cytometry assay results are depicted in Figure 5C,D, and consistent with the laser confocal findings, SiPc–NO@RGD NPs were more effectively internalized by 4T1 and MCF-7 cells.

### 3.6. In Vitro NO Release

**Figure 6:**
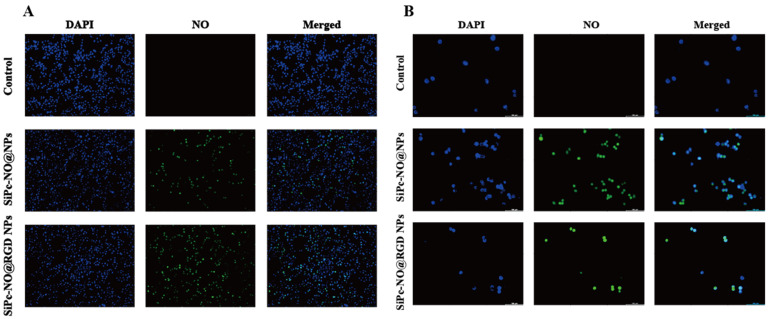
(**A**) SiPc–NO self-assembled nanoparticles affect NO content in 4T1 cells. (**B**) SiPc–NO self-assembled nanoparticles affect NO content in MCF-7 cells.

Previous experiments have verified that SiPc–NO, SiPc–NO@NPs, and SiPc–NO@RGD NPs can effectively produce NO in solution, but intracellular NO release needs to be further examined. DAF-FM-DA is a novel fluorescent probe, which can penetrate cells through cell membranes and transform into the impermeable DAF-FM through interaction with intracellular esterases. It is extensively utilized in the realm of NO detection. DAF-FM fluorescence is initially weak, but it can be intensified to generate robust fluorescent signals upon interaction with NO, and it exhibits high sensitivity, with an excitation wavelength of 495 nm and an emission wavelength of 515 nm [43,44]. As depicted in Figure 6, following the induction of SiPc–NO self-assembled nanoparticles, the intracellular NO level in the experimental group showed a significant increase. The green fluorescence of cells treated with SiPc–NO@RGD NPs was notably stronger compared to SiPc–NO@NPs, aligning with the results of cellular uptake assay experiments. This enhancement can be attributed to the RGD targeting moiety on SiPc–NO@RGD NPs, which specifically binds to the αvβ3 integrin receptor on the cell membrane, resulting in increased cellular uptake and intensified intracellular fluorescence.

### 3.7. Reactive Oxygen Species Generation Ability

**Figure 7:**
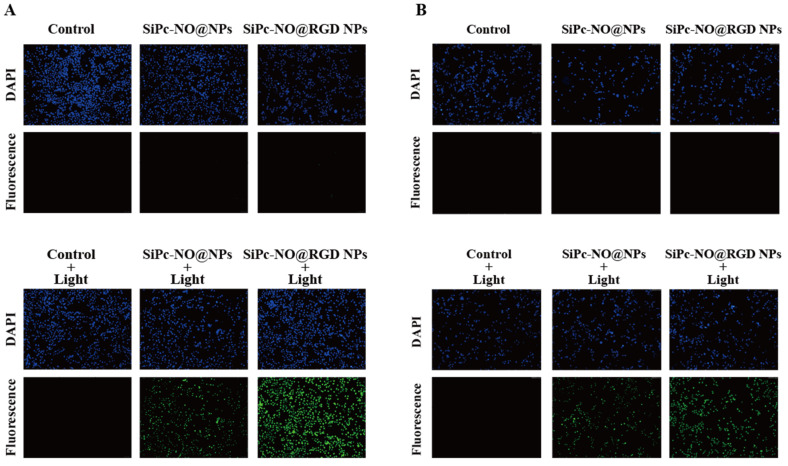
(**A**) SiPc–NO self-assembled nanoparticles affect reactive oxygen species content in 4T1 cells. (**B**) SiPc–NO self-assembled nanoparticles affect reactive oxygen species content in MCF-7 cells.

The capacity of photosensitizers to generate reactive oxygen species inside cells is crucial in assessing their photodynamic therapeutic effects. Hence, the intracellular production of reactive oxygen species by SiPc–NO self-assembled nanoparticles was investigated utilizing 2′7′-dichlorofluorescein diacetate (DCFH-DA) as a probe for reactive oxygen species [45,46]. The DCFH-DA assay principle is as follows: once inside the cell, DCFH-DA molecules undergo enzymatic hydrolysis, converting them to DCFH molecules. Due to the impermeability of the cell membrane, DCFH cannot freely diffuse outside the cell but remains confined within the cell. Under the influence of intracellular reactive oxygen species, DCFH molecules undergo oxidation and transform into fluorescent DCF molecules. These DCF molecules emit green fluorescence when exposed to specific wavelengths of light. By observing the fluorescence intensity of DCF within the cell, one can determine the level of intracellular reactive oxygen species and evaluate the extent of oxidative stress in the cell. The results are displayed in Figure 7. In the absence of irradiation, no green fluorescence was observed in either the control or experimental groups. Obvious green fluorescence was observed in cells treated with DCFH-DA and SiPc–NO self-assembled nanoparticles upon laser irradiation. The level of fluorescence produced correlated with the outcomes of the cellular uptake experiments. Notably, the production of reactive oxygen species by SiPc–NO@RGD NPs was significantly higher compared to SiPc–NO@NPs.

### 3.8. Cell Scratch Assay

**Figure 8:**
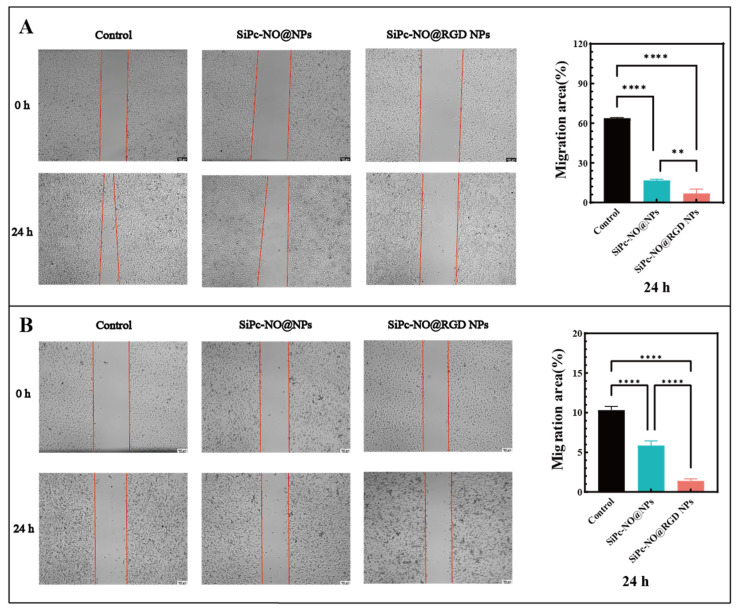
(**A**) SiPc–NO self-assembled nanoparticles inhibit the healing ability of 4T1 cells. (**B**) SiPc–NO self-assembled nanoparticles inhibit the healing ability of MCF-7 cells. Significance identification: **, *p* < 0.01; ****, *p* < 0.0001.

Migration and invasion of tumor cells are significant contributors to cancer development, progression, metastasis, and recurrence [47,48]. Scratch experiments were conducted to further investigate the impact of the self-assembled nanoparticles on the migration of 4T1 and MCF-7 cells. The findings are illustrated in Figure 8, showing a general healing trend across all groups. SiPc–NO self-assembled nanoparticles effectively inhibited cell migration in both cell types, with the healing rate of SiPc–NO@RGD NPs being lower than that of the SiPc–NO@NP group in both cases. The healing rate of SiPc–NO@RGD NPs compared to SiPc–NO@NPs decreased by 2.18-fold in 4T1 cells and 4.21-fold in MCF-7 cells. This indicates that the prepared SiPc–NO@RGD NPs have a strong inhibitory effect on the migration ability of both cells. The data from both groups demonstrated statistical significance (*p* < 0.01).

## 4. Discussion

In tumor treatment, photodynamic therapy offers advantages such as high tumor selectivity, effective enrichment, minimal damage to normal tissues, and strong reproducibility. It has emerged as a research hotspot in the treatment of malignant tumors and has demonstrated remarkable clinical results in addressing superficial tumors [49].

A novel approach to the targeted delivery of self-assembled nanomedicines utilizing photodynamic synergistic treatment and NO was presented. In this study, the NO donor and the photosensitizer silicon phthalocyanine with ester bonds were self-assembled into nanoparticles in water. The morphology of SiPc–NO@RGD NPs was examined using transmission electron microscopy (TEM). SiPc–NO@RGD NPs were spherical, with an average diameter of approximately 90 nm and a hydrodynamic size of about 100 nm, demonstrating high dispersity in water. The encapsulation rate and drug loading capacity of the self-assembled nanoparticles reached up to 80% and 60%, respectively. The drug loading capacity exceeds ten times that of traditional liposome delivery systems, enabling effective delivery of NO. Alongside preparative characterization, optical and chemical characterization of SiPc–NO self-assembled nanoparticles was conducted.

In vitro experiments showed that SiPc–NO@RGD NPs were significantly phototoxic to 4T1 and MCF-7 breast cancer cells. The self-assembled nanoparticles were low-cytotoxic to 4T1 and MCF-7 cells without light. At the same time, the IC_50_ value was 0.5311 μM and 0.2975 μM with light, indicating that the nanoparticles can be activated by light specifically at the tumor site. Laser confocal microscopy revealed that SiPc–NO@RGD NPs could be taken up by 4T1 and MCF-7 cells and efficiently generated ROS and NO intracellularly. SiPc–NO@RGD NPs significantly inhibited the healing ability of 4T1 and MCF-7 cells. Overall, this study offers new insights into gas therapy, photodynamic synergistic therapy, and self-assembled nanoparticles.

## 5. Conclusions

In the present study, SiPc–NO was prepared as self-assembled nanoparticles using the nanoprecipitation method. The optimized nanoformulations were investigated and evaluated in terms of appearance and morphology, particle size, PDI, zeta potential, encapsulation rate, drug loading capacity, and initial stability. The prepared SiPc–NO@RGD NPs exhibited smaller particle sizes with a higher drug loading capacity, and stability test studies demonstrated stable results for the measured parameters. At the cellular level, the nanoparticles displayed low toxicity to cancer cells under non-photo-irradiated conditions. The SiPc–NO@RGD NPs were photo-responsive and targeted, producing large amounts of NO and reactive oxygen species in the cells, significantly inhibiting the viability and migration of breast cancer cells. Additionally, there are several elements that can be improved in this study: (1) Due to time constraints, it was not possible to examine the anti-tumor effect of nanoparticles in vivo; experimental studies on their tumor targeting and photodynamic aspects in vivo can be supplemented in future research; (2) the mechanism of NO and reactive oxygen species synergy should be investigated through corresponding experiments to explore their biological functions and interactions in cells; (3) photosensitizers with better water solubility can be selected to construct amphiphilic drug–drug conjugates to enhance the anti-tumor effect and stability of self-assembled nanoparticles. If this can be achieved, effective delivery of NO and photosensitizers without using any excipients will be possible. In summary, SiPc–NO@RGD NPs serve as highly targeted drug carriers for NO and photosensitizers, improving anti-tumor activity and safety.

## Figures and Tables

**Table 1:** IC_50_ of SiPc–NO self-assembled nanoparticles.

NPs	IC_50_ (μM)	
	4T1	MCF-7
SiPc–NO@NPs	0.9141	0.6394
SiPc–NO@RGD NPs	0.5311	0.2975

## Data Availability

Most included studies are publicly available via open access on journal websites. Additional data and code are available upon request for privacy reasons.

## Supplementary Materials

The following supporting information can be downloaded at: https://www.mdpi.com/article/10.3390/pharmaceutics16091166/s1, Figure S1. 1H NMR spectrum of SiPc-NO in DMSO (600 MHz). Figure S2. ^13^C NMR spectrum of SiPc-NO in CDCl_3_ (600 MHz). Figure S3. IR spectrum of SiPc-NO. Figure S4. HRMS spectrum of SiPc-NO. Figure S5. Reactive oxygen determination of SiPc-NO self-assembled nanoparticles (A) SiPc-NO (B) SiPc-NO@NPs.

## Abbreviations

The following abbreviations are used in this manuscript:

CCK-8	Cell Counting Kit-8
DMSO	Dimethyl sulfoxide
DMAP	4-dimethylaminopyridine
DTDP	3,3′-dithiodipropionic acid
DPBF	1,3-diphenylisobenzofuran
DSPE-PEG_2K_	Distearoyl phosphoethanolamine-PEG2000
DSPE-PEG_2K_-RGD	Distearoyl phosphoethanolamine-PEG2000-Arg-Gly-Asp
DL	Drug loading
DCF	2′,7′-dichlorofluorescin
DAF-FM	3-amino,4-aminomethyl-2′,7′-difluorofluorescein, diacetate
EDC	1-ethyl-3-(3-dimethylaminopropyl)carbodiimide
EE	Encapsulation efficiency
EtOAc	Ethyl acetate
HRMS	High-resolution mass spectrometry
HPD	Hematoporphyrin derivatives
IR	Infrared spectroscopy
IC_50_	Half-maximal inhibitory concentration
IARC	International Agency for Research on Cancer
MCF-7	Human breast cancer MCF-7 cells
m-CPBA	3-chloroperoxybenzoic acid
NMR	Nuclear magnetic resonance
NO	Nitric oxide
NPs	Nanoparticles
OD	Optical density
PDT	Photodynamic therapy
PS	Photosensitizer
PBS	Phosphate-buffered saline
RGD	Arg-Gly-Asp
ROS	Reactive oxygen species
SiPc	Porphyrin silicon
SAN	Self-assembled nanoparticles
UV	Ultraviolet
4T1	Mouse breast cancer cells
