# Supplementary material for: Self-Assembled Nanoparticles of Silicon (IV)–NO Donor Phthalocyanine Conjugate for Tumor Photodynamic Therapy in Red Light
Source: Pharmaceutics. 2024 Sep 4;16(9):1166. doi: 10.3390/pharmaceutics16091166 (PMC11435187; doi:10.3390/pharmaceutics16091166)
Supplement: Supplementary file 1 [file pharmaceutics-16-01166-s001.zip › pharmaceutics-3146051-supplementary.pdf]

Supplementary File

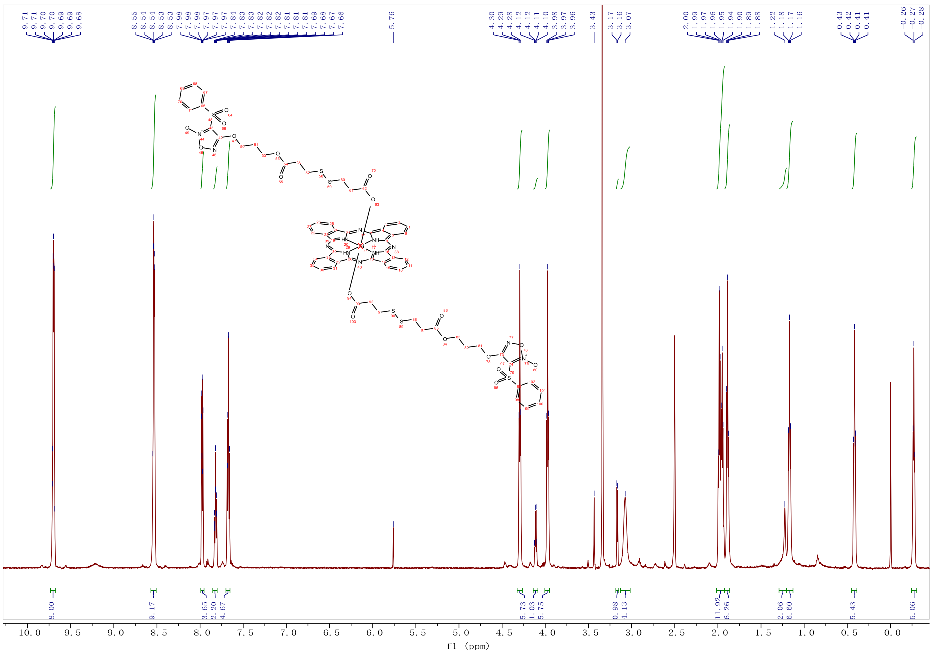

Figure S1. ^{1}H NMR spectrum of SiPc-NO in DMSO (600 MHz).

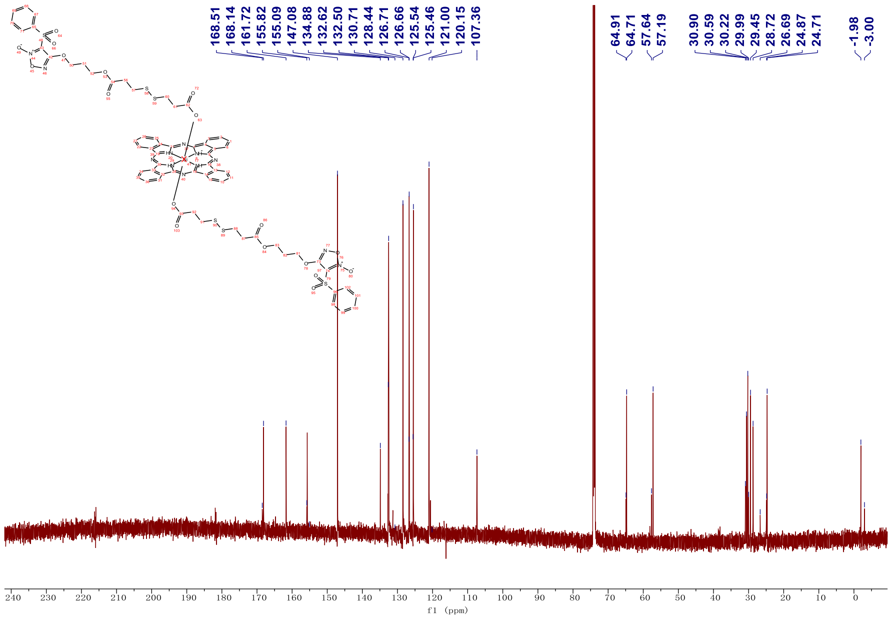

Figure S2. ^{13}C NMR spectrum of SiPc-NO in $CDCl_3$ (600 MHz).

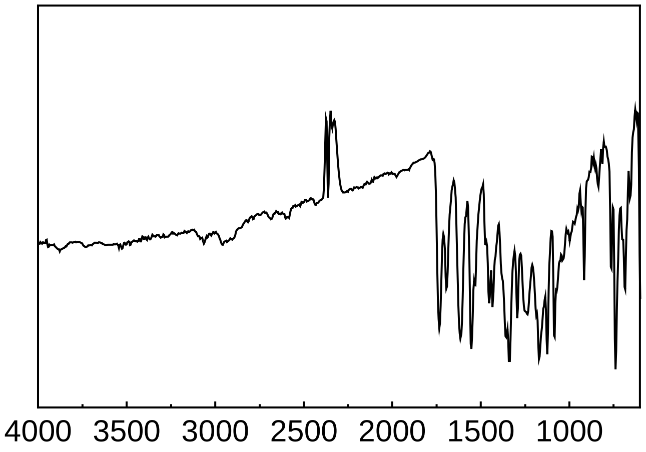

Figure S3. IR spectrum of SiPc-NO.

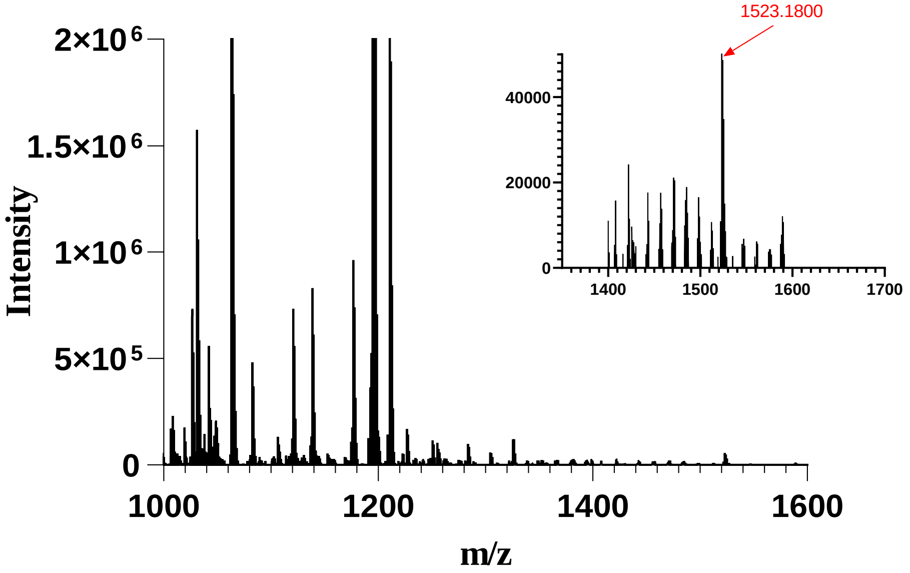

Figure S4. HRMS spectrum of SiPc-NO.

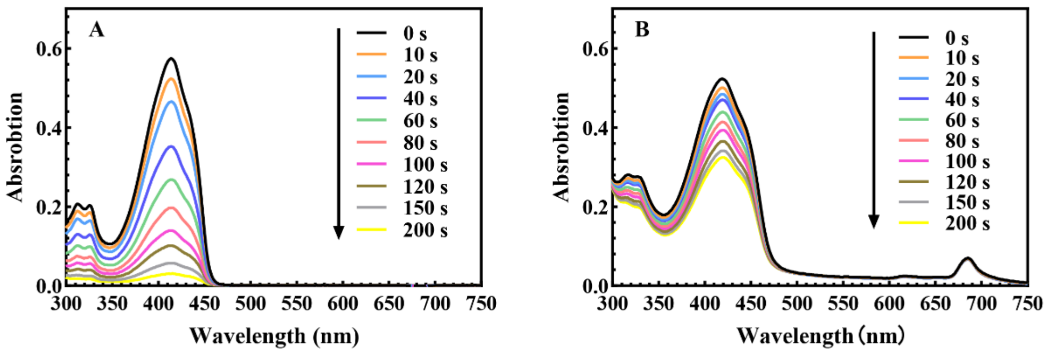

Figure S5. Reactive oxygen determination of SiPc-NO self-assembled nanoparticles（A）SiPc-NO（B）SiPc-NO @ NPs.
